# A Curious Case Report: Should a Clinician Be Worried About Bone Resorption Under a Chin Augmentation Site?

**DOI:** 10.7759/cureus.37041

**Published:** 2023-04-02

**Authors:** Kavya Shankar Muttanahally, Aditya Tadinada

**Affiliations:** 1 Oral and Maxillofacial Radiology, Department of Adult Restorative Dentistry, University of Nebraska Medical Center, Lincoln, USA; 2 Oral and Maxillofacial Radiology, University of Connecticut, Farmington, USA

**Keywords:** chin augmentation, cone-beam computed tomography (cbct), microgenia, chin deformity, silastic implant

## Abstract

Oftentimes patients are concerned about their facial esthetics and appearance. To achieve the desired look, patients have several augmentation procedures available to them. Appearance of the chin and its morphology plays a crucial role in facial aesthetics. It is an important anatomic component that not only helps in the definition of the jawline and face but is also required from a functional aspect. It is not uncommon in the field of plastic surgery for patients to undergo chin reconstruction and recontouring due to the chin deformities such as microgenia and jaw asymmetry. The treatment options mainly depend on the degree of the defect and the desired functional and esthetic requirements. Along with surgical augmentations like implants and osseous genioplasty, soft tissue augmentations like injectables are also gaining popularity. These procedures can lead to complications like many other augmentation procedures. If proper follow-up is not done on these patients, the complications can cause potential damage to vital structures in the vicinity. This case report shows a patient who underwent a chin augmentation procedure with a silastic implant and never had a follow-up and is now at risk of severe resorption of the underlying bone.

## Introduction

An aesthetically pleasing face depends on multiple factors including the proportions of facial structures and their symmetry. Chin is a crucial anatomic component to maintain harmony through its definition and projection, and for maintaining a pleasing jawline. Often, the chin can be deformed/deficient, and it is called microgenia. It is the most common type of chin deformity, significantly noticed in the sagittal plane. This deformity results from two factors, either from soft-tissue atrophy or retrusion of the mandible [1,2]. The lower third of the face plays a significant role in plastic surgery. Reconstruction and recontouring of the chin, referred to as chin augmentation is a common treatment option for correcting chin deformities [3-5]. The treatment depends on the type and extent of the deformity and the required esthetic and functional outcome. Chin augmentation may be performed with injectables, implants, or osseous genioplasty [6].

Other alternative methods for the chin augmentation procedure include osteotomies and fat/filler injections. To carry out the chin augmentation procedure, alloplastic materials such as silastic (solid silicone), porous polyethylene, and polytetrafluoroethylene have been used [7]. Currently, silastic/silicone is the most popularly used material for implants as it causes minimal soft tissue adhesion, making it less complicated while inserting and removing the implants when required.

There are certain post-surgical complications, both long term and short term that occur with such augmentation procedures [4]. Around 85% to 90% of postsurgical patients are satisfied after genioplasty [7,8]. However, with any surgery, there can be complications, as the use of implants can also cause complications. There are numerous complications that can occur during implant surgical procedures that mainly include infections, migration, oral incompetence, implant displacement, and damage of the mentalis muscle and mental nerve. Additionally, post-operative secondary bone resorption could cause potential damage to the roots, injure nerves and withhold chin growth in young growing patients [8-10].

The most disturbing complication is bone resorption underneath the augmentation procedure. The reason for this complication is due to the pressure exerted on the underlying bone with chin movements that are continuous while chewing, talking, and smiling. It is a dynamic region along with the lip and mouth movements. The other etiology for bony erosion is improper positioning: placing the implant too high over the thin cortical bone. The pressure is due to bulky implants [11,12]. In most patients, bony erosion is frequent and is evident in radiographs. In this article, we present a case report of a patient at risk of bony erosion following chin augmentation with a silastic implant.

## Case presentation

**Figure 1:**
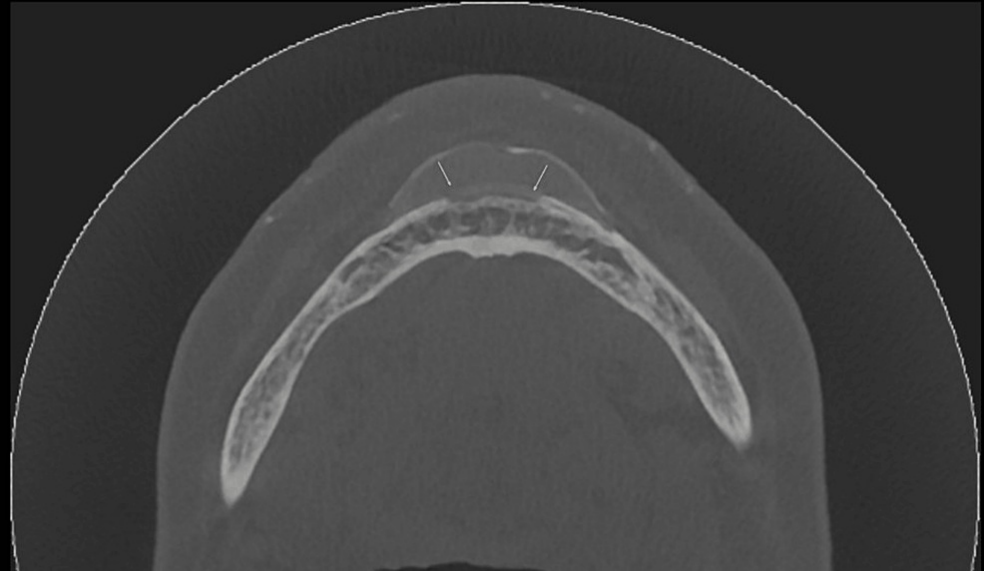
The axial view of the cone-beam computed tomography (CBCT) showing the bony erosion of the buccal cortex in the mandibular anterior region (arrows).

**Figure 2:**
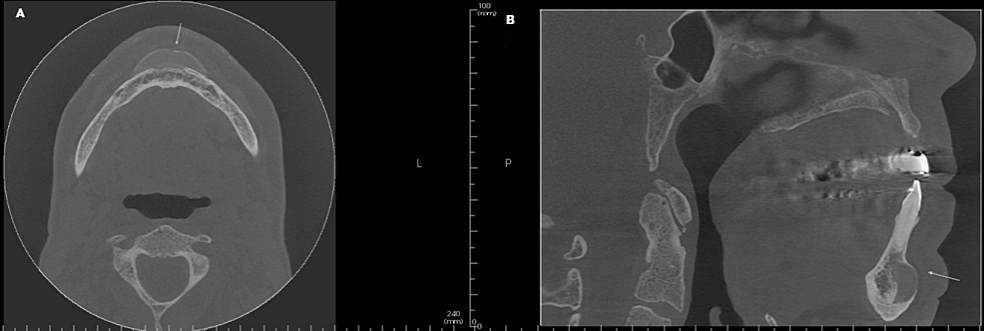
Cone-beam computed tomography (CBCT) scan showing (axial and sagittal views) thin radiopaque structure protruding from the chin (arrows). A: Axial view showing the thin radiopaque structure. B: Sagittal view showing the thin chin implant protruding from the chin.

**Figure 3:**
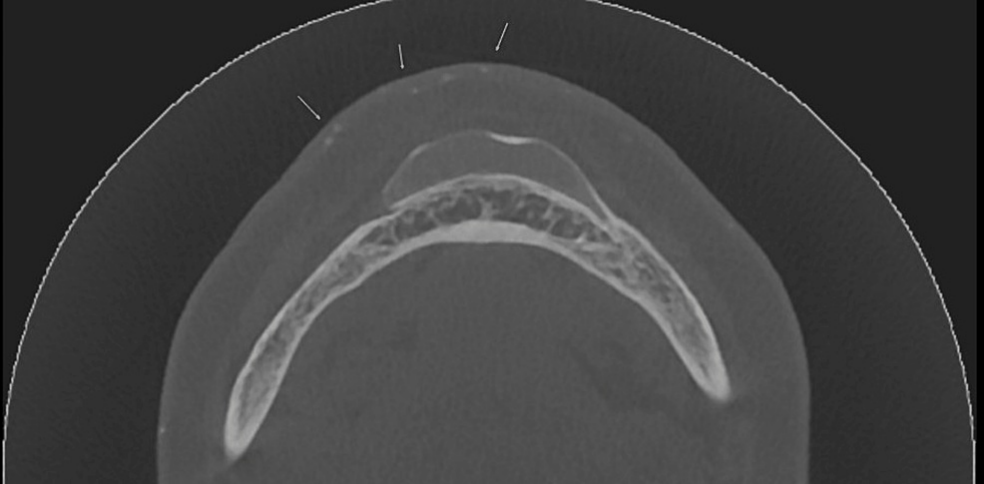
Axial view of cone-beam computed tomography (CBCT) showing multiple tiny radiopaque flecks in soft tissue overlying the chin.

**Figure 4:**
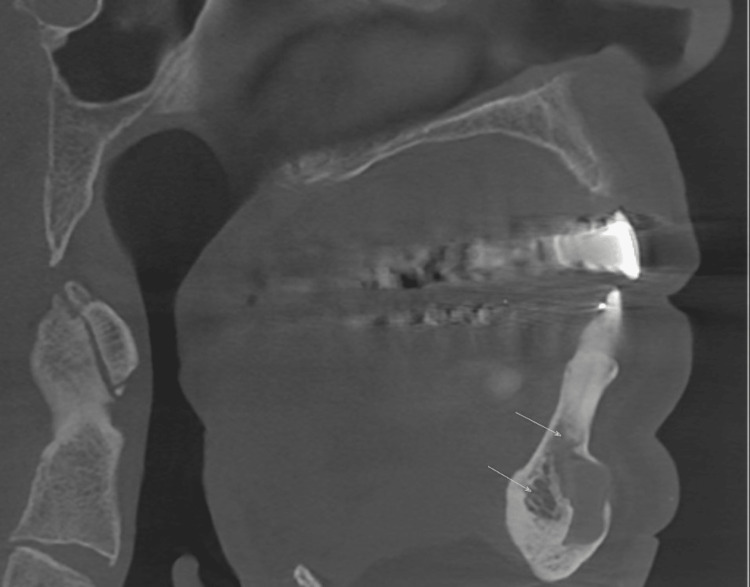
Sagittal section of cone-beam computed tomography (CBCT) showing the bone resorption and periapical radiolucency in the mandibular anterior teeth.

A 61-year-old female patient was referred by a prosthodontic practice for a panoramic radiograph for routine evaluation and implant treatment planning. The panoramic radiograph was acquired using Planmeca (Hoffman Estates, IL, USA) Promax Digital 2D S3 PAN Panoramic X-Ray Unit using parameters 70 kVp and 6 mA. The panoramic image showed a subtle radiolucency and was symmetric in the anterior region of the mandible. Due to the ghost image of the C-spine in the anterior mandibular region, the border and full the extent of the entity was not well visualized. Cone-beam computed tomography (CBCT) scan was recommended to further evaluate the anterior mandibular region as the panoramic radiograph did not reveal the required information for making an accurate diagnosis. The CBCT was acquired with a J Morita (Irvine, CA, USA) Accuitomo 170 unit with a 140X100 mm field of view (FOV), 90 kVp and 7 mA. The Dicom data was analyzed using a third-party CBCT reconstruction program, Invivo-6 (Anatomage Inc., San Jose, CA, USA). The axial view (Figure 1) and sagittal view (Figure 2) of CBCT showed a uniform, well-defined, radiopaque line extending from the outer cortex of the chin/symphysis mimicking the appearance of a benign lesion in the anterior mandible. We also observed multiple tiny radiopaque flecks noted in the soft tissue overlying the chin, likely collagen or botulinum fillers (Figure 3). The scan also revealed bony erosion of the outer cortex of the mandible. In terms of the spatial location of the lesion, three-dimensional imaging revealed that the radiolucency was located on the outer aspect of the cortical plate and apical to the mandibular anterior teeth (Figure 4). A critical feature that was not evident on the two-dimensional panoramic radiograph. With information from the CBCT scan, a radiographic diagnosis of augmentation in the chin area consistent with a silastic implant with marked erosion of the buccal cortex was made. Surprisingly, there were no obvious clinical findings noted and the patient reported no discomfort and was asymptomatic. The patient was then referred to an endodontist for evaluation of the anterior mandibular teeth and then to her plastic surgeon for evaluation of the chin implant. This is one of the case reports where there is marked erosion of the underlying bone with the patient exhibiting no signs and symptoms.

## Discussion

Genioplasty is a surgical procedure done for chin correction in terms of shape, size, and functionality. In order to do so, either the osseous correction or augmentation with injectables and implants is done. Currently, chin augmentation with a silastic implant is a very common cosmetic procedure [12]. Chin augmentation surgery is frequently carried out by oral and maxillofacial surgeons and plastic surgeons. There were 18,136 chin surgeries performed in the year 2014 based on a report by the American Society of Plastic Surgeons [12,13].

Bone resorption is a common risk associated with chin implants. A favorable factor for the success or failure of a chin implant is the location of the implant in the chin. Unlike endosseous implants, chin implants are situated in the sub-periosteal region, where there is a potential risk of damaging the underlying bone due to the pressure exerted by the implant on the bone. Additionally, the implant material also plays a crucial role. If the erosion of the bone progresses, it can affect the teeth or cause nerve paresthesia if it pushes on the mental nerve. When such complications are observed, implant removal is recommended [14]. The dentist, the oral radiologist, and surgeons should be aware of these newer procedures that are gaining significant popularity and must learn to recognize any associated complications for appropriate diagnosis and triage.

There are mainly two classifications used for chin implant bone erosion. Robinson’s [14] classification, is a radiographic classification based on lateral skull radiographic views (Table 1). It is based on the amount of resorption (depth) compared with the size of the implant used. Guyuron et al.’s [15] classification is primarily based on the depth of bone invaded (Table 1). We classify our current case report under Guyuron classification 2, where there is cortical bone resorption under the implant.

**Table 1:** Robinson and Guyuron classification systems of bone resorption

Robinson Classification system of bone resorption [14]
1. Immediate resorption up to third of the added implant dimension, or approximately 3mm
2. Resorption between a third and a half of the added implant dimension, or approximately 3-5mm
3. More than 50% of the implant dimension, or 5mm
Guyuron classification system of bone resorption [15]
1. Cortical bone resorption
2. Bone resorption up to 3 mm
3. Bone resorption of 3-5 mm
4. Bone resorption over 5 mm

Polo, in 2018 [16], reported bone resorption in three out of four patients who had undergone chin implant surgery. These patients had panoramic and lateral cephalometric radiographs taken for their routine orthodontic evaluation. Although one of the patients had normal symphyseal contour, the rest of the three patients had significant bone resorption beneath the chin implants. One of the patients even had magnetic resonance imaging (MRI) and computerized tomography (CT) due to severe resorption and potential risk of nerve damage. These were incidental findings like our case where the actual intent of the CBCT scan was for dental implant treatment planning in the mandibular posterior edentuolus region. 

The general dentist/clinician should look out for signs of bone resorption, alteration in the periapical regions, and any abnormal sensation in the area of the chin implants and adjoining areas. A periodic follow-up is helpful in early detection of any potential complication. Imaging with a low-dose CBCT scan would provide the oral radiologist and the clinician to determine the borders of the implant and the underlying bone and will help quantify the magnitude of the bone resorption at the mandibular facial/buccal cortex in a more precise manner [17]. Recognizing and reporting these findings to the respective clinicians and surgeons enables the establishment of a more appropriate medical and surgical follow-up resulting in favorable outcomes, especially for a patient who is in the early stages of bone resorption.

There are potential possible sequelae that can occur due to the bone resorption in the chin area. Dentists are mainly concerned about the tooth vitality, tooth fracture and even the mandibular cortication fracture [18]. The other effects of chin resorption due to implant failure are reduction in the prominence of the chin and ultimately facial disfigurement [18,19]. If the bone resorption under the chin implant is noticed in the early stages and measures like correction of implant location or when necessary, removal of the implant, would significantly avoid the damage to the vital structures as well as avoid disfigurement of the face [20]. 

## Conclusions

There are both short-term and long-term complications associated with the chin augmentation procedure. These patients require periodic follow-up clinical and radiographic examinations in order to identify any complications for early diagnosis and triage.
